# Widespread evidence for elephant exploitation by Last Interglacial Neanderthals on the North European plain

**DOI:** 10.1073/pnas.2309427120

**Published:** 2023-12-04

**Authors:** Sabine Gaudzinski-Windheuser, Lutz Kindler, Wil Roebroeks

**Affiliations:** ^a^MONREPOS Archaeological Research Centre and Museum for Human Behavioural Evolution (LEIZA), Neuwied 56567, Germany; ^b^Institute of Ancient Studies, Pre- and Protohistoric Archaeology, Johannes Gutenberg-University Mainz, Schönborner Hof, Mainz 55116, Germany; ^c^Faculty of Archaeology, Leiden University, 2300 RA Leiden, The Netherlands

**Keywords:** Neanderthals, Last Interglacial, group size, evolution of cooperation, megaherbivores

## Abstract

Neanderthals hunted and butchered straight-tusked elephants, the largest terrestrial mammals of the Pleistocene, in a lake landscape on the North European plain, 125,000 years ago, as recently shown by a study of the Last Interglacial elephant assemblage from Neumark-Nord (Germany). With evidence for a remarkable focus on adult males and on their extended utilization, the data from this location are thus far without parallel in the archaeological record. Given their relevance for our knowledge of the Neanderthal niche, we investigated whether the Neumark-Nord subsistence practices were more than a local phenomenon, possibly determined by local characteristics. Analyzing elephant remains from two other Last Interglacial archaeological sites on the North European plain, Gröbern and Taubach, we identified in both assemblages similar butchering patterns as at Neumark-Nord, demonstrating that extended elephant exploitation was a widespread Neanderthal practice during the (early part of the) Last Interglacial. The substantial efforts needed to process these animals, weighing up to 13 metric tons, and the large amounts of food generated suggest that Neanderthals either had ways of storing vast amounts of meat and fat and/or temporarily aggregated in larger groups than commonly acknowledged. The data do not allow us to rule out one of the two explanations, and furthermore both factors, short-term larger group sizes as well as some form of food preservation, may have played a role. What the data do show is that exploitation of large straight-tusked elephants was a widespread and recurring phenomenon amongst Last Interglacial Neanderthals on the North European plain.

## Significance

We have recently learned that around 125,000 years ago, hunting of straight-tusked elephants, the largest terrestrial mammals of the Pleistocene, was part of the Neanderthal behavioral repertoire, for several dozens of generations. This knowledge is based on data from one lake-side location in northern Europe only, and hence possibly of limited value for our knowledge of the Neanderthal niche. This new study presents data from two other, contemporaneous sites on the North European plain, demonstrating that elephant exploitation was a widespread phenomenon there. The sheer quantities of food generated by the butchering activities, aimed at extensive exploitation of the carcasses, suggest that Neanderthals had some form of food preservation and/or at least temporarily operated in larger groups than commonly acknowledged.

It is now well-established that Neanderthals were successful hunters of a wide range of prey animals (1–3). Successful hunting depended on knowledge of the behavior of animals and of their environment, efficient weaponry, and careful planning of activities, and, varying with prey types, well-coordinated cooperation between members of local groups. The killing of a prey animal initiated a range of processing activities, from initial butchery to the preparation of organs, meat, and fat for consumption and possibly storage and transport. It is for these reasons that the study of subsistence practices can inform about less tangible aspects of the niche of Pleistocene humans, such as cooperation, local group size, mobility, and social organization. Subsistence activities also included foraging for plant foods. In fact, the exploitation of plants was a deep-rooted, widespread, and important part of Neanderthal subsistence, (e.g., ref. 4), but the animal component of their diet is simply better preserved and hence more visible in their archaeological record.

The wide array of Neanderthal prey animals known through archaeozoological studies includes small and fast-moving ones, like rabbits and birds (5, 6), carnivores and dangerous omnivores such as bears (7, 8), large herbivores, e.g., bovids and horses, and megaherbivores (body mass > 1,000 kg) such as rhinoceros. The latter are well documented at various Middle and Late Pleistocene sites, e.g., at Biache-Saint-Vaast, France (9), with an age of around 200,000 y (200 ka) and at the Last Interglacial (Eemian) site Taubach, Germany (8). A recent study (10) demonstrates that megaherbivore prey also included straight-tusked elephants, the largest terrestrial mammals of the Pleistocene, uniquely documented at the Eemian site Neumark-Nord 1, Germany. This site has yielded the world’s richest *Palaeoloxodon (P.) antiquus* assemblage, recovered during long-term rescue excavations in Last Interglacial lake-shore deposits, exposed in a large lignite strip-mining quarry.

Our recent analysis of this *P. antiquus* assemblage, with a Minimum Number of Individuals (MNI) of 57, shows that Neanderthals had primary access to fresh carcasses, and intensively processed these in similar ways, during minimally 2,000 y in the early part of the Last Interglacial (10). The peculiar age and sex profile of the assemblage, with a lack of juvenile individuals and a strong bias toward older male adults, differs substantially from that of natural mortality patterns of modern African elephants (11, 12) and resembles mortality patterns created by ivory poaching (13). The Neumark-Nord hominins were targeting very specific age classes of the elephant population in the lake area, with a remarkable focus on adult males.

As detailed elsewhere (10), the cut mark distribution pattern, with a frequent presence of cut marks on the diaphyses of long bones, demonstrates access to fresh carcasses, in line with data obtained in studies of recent elephant assemblages (14). Of further relevance here, Haynes and Krasinski (14) identified three types of elephant carcass exploitation, respectively, satisficing, extended, and maximized utilization. In satisficing utilization, butchers were satisfied with taking only parts of the carcass, such as tusks and most of the muscle masses, activities that left no cut marks on the large limb bones. In extended utilization, all meat was stripped from the largest limb bones, resulting in clearly visible cut marks on the diaphyses (14). Extended utilization also created very specific cut mark patterns, such as “parallel or subparallel fields at right angles or diagonal to a bone’s long axis” (14), likewise identified at Neumark-Nord (10). The occasional presence of cut marks on the interior side of Neumark-Nord elephant ribs, an indication of maximized carcass utilization *sensu* Haynes and Krasinski (14), underlines the “extended utilization” diagnosis for this assemblage (10).

The unusually high amount of adult and old male individuals was probably related to the strong sexual dimorphism of *P. antiquus*, with males substantially taller and with a body mass more than twice that of females (15, 16). Among extant elephants, male adults are usually solitary or in small all-male groups during feeding and traveling and stay apart from mixed herds of females and young. If that was also the case during the Last Interglacial, the largest “calorie packages” would have roamed the landscape in relative isolation, without the extra vigilance against hunters provided by being in a herd. Targeting these males, of up to 13 metric tons in weight (16), would have yielded high returns for a comparatively low risk, though it is obvious that killing such animals would have been a risky enterprise that required cooperation among multiple hunters. Prior to the widespread use of firearms, traditional elephant hunting was a dangerous high-cost activity, in the ethnographic record consistently associated with prestige seeking of hunter-gatherers (17).

The extended utilization [*sensu* (14)] of the carcasses yielded large amounts of food: As detailed in a previous study, the processing of a 10 metric tons individual, not the largest one from Neumark-Nord for example, would have provided more than 2,500 daily portions of 4,000 Kcal for adult Neanderthals, consisting of a “protein-poisoning safe” mixture of fat and protein (ref. 10 and *SI Appendix*, Text 2). The sheer quantities of food imply large groups of consumers, possibly in the form of temporary aggregations of various local groups from within the wider area, and/or point to the existence of cultural ways of storage of meat and fat, both socially and cognitively important findings (see ref. 18 and *Discussion*). However, how far can the Neumark-Nord findings be generalized? The Neumark-Nord evidence constitutes a unique case thus far. This well-preserved archaeological and paleontological record was created in a resource-rich landscape of shallow lakes and pools, which could entail that specific characteristics of this lake area enabled a specific local type of foraging that was not more widely distributed during the Last Interglacial. The local scale of the data hence might have limited relevance for our understanding of Neanderthal behavior on a wider scale.

**Fig. 1.**
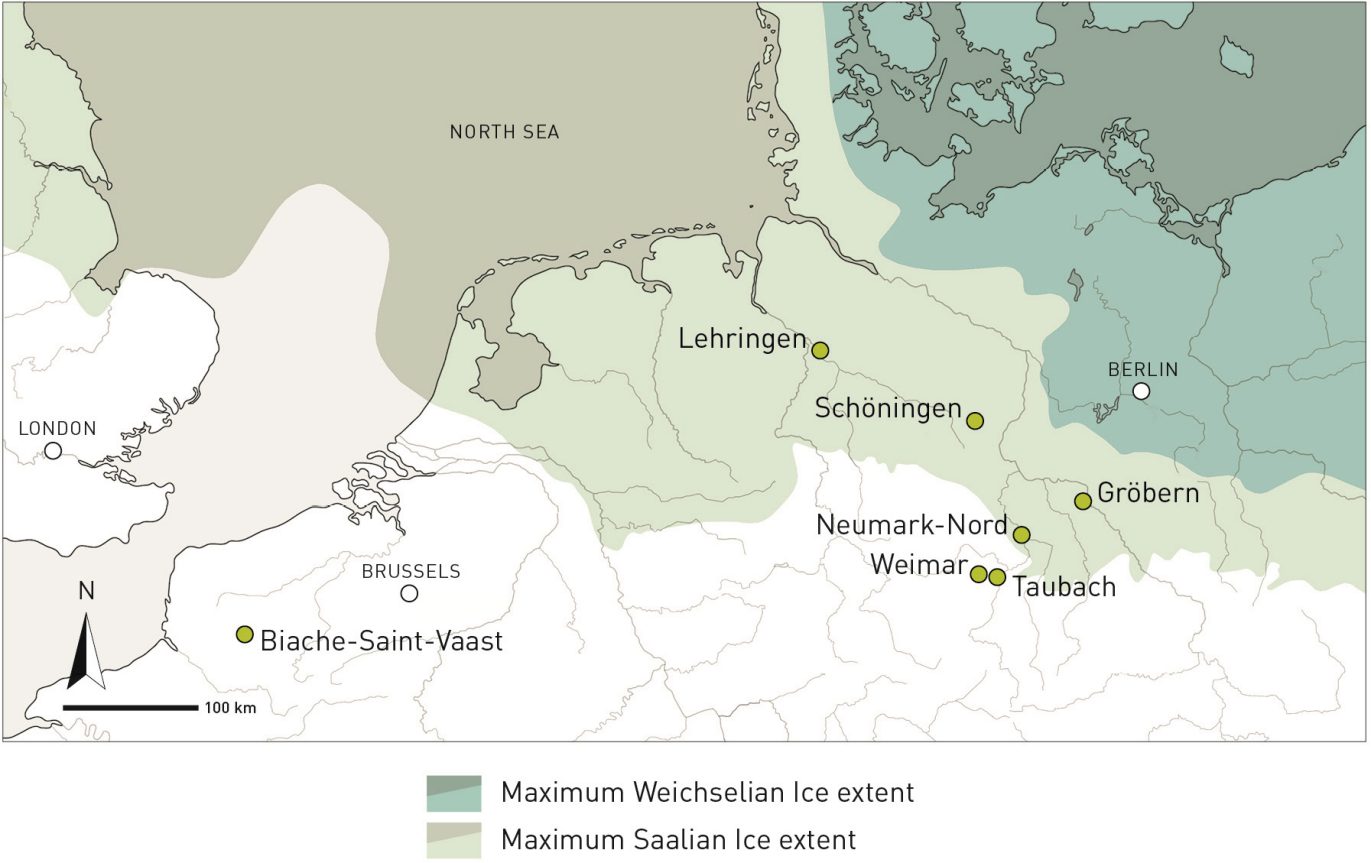
Location of Neumark-Nord, Gröbern, Taubach, and other sites on the northern European plain, relative to the maximum extent of the Saalian and Weichselian glaciers.

Thus far, we have no comparative material from other sites of similar quality and quantity to address this question straightforwardly. The area in which Neumark-Nord is situated (Fig. 1) is a ~100 to 200 km wide zone between the maximum extension of the penultimate [Saalian, Marine Isotope Stage (MIS) 6] and the last (Weichselian, MIS2) glaciation of the northern European plain. This zone has a relatively large number of Last Interglacial archaeological sites, with contemporaneous deposits elsewhere in Europe very rarely accessible only (19). Traces of Last Interglacial Neanderthal presence are preserved in this zone in MIS 6 glacial-ice-carved depressions, uniquely exposed during large-scale brown coal strip mining, enabling discovery of Last Interglacial landscapes through their destruction. Just south of the maximum extent of the Saalian ice sheet, there are also some important Last Interglacial sites in the travertine sequences of Central Germany (Fig. 1).

To establish whether the Neumark-Nord elephant data are relevant for our understanding of Neanderthal behavior in a wider area, one needs records of the same age and with a good faunal preservation. We indeed did identify two early Last Interglacial archaeological sites from the above-mentioned area, the only two other Eemian sites known with well-preserved elephant remains recovered in association with lithic artifacts, and from which sufficient material is still available for study. These sites are situated at, respectively, 55 km northeast and 54 km southwest of Neumark-Nord: They are Gröbern and Taubach (Fig. 1).

## Gröbern.

**Fig. 2.**
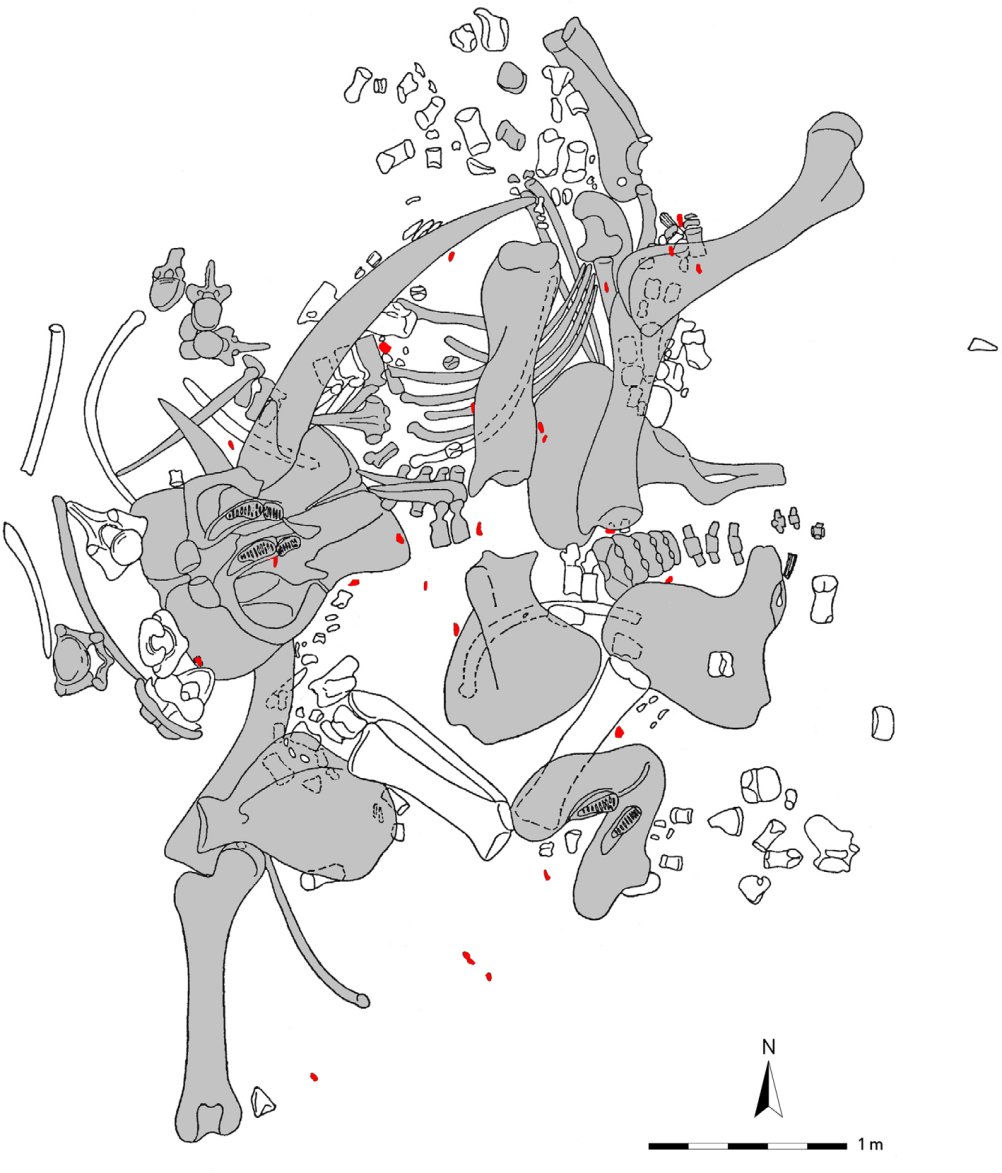
Spatial distribution of the skeletal remains of the Gröbern *P. antiquus* skeleton. In gray, skeletal elements analyzed in this study. In red, flint artifacts (Redrawn after ref. 21)

In 1987, a virtually complete *P. antiquus* skeleton was uncovered during quarrying activities in a lignite mine at Gröbern, near Leipzig. The remains were present on the sandy shores of a Last Interglacial lake, embedded in the limnic deposits infill of a Saalian (MIS 6) glacial basin (20), and were documented in a rescue excavation by the Landesmuseum für Vorgeschichte at Halle (21) (Fig. 2). The embedding sediments formed at the transition between pollen assemblage zone (PAZ) IVb (hazel–yew–lime) and V (the hornbeam phase) of the Last Interglacial, *sensu* Menke, and Tynni (22). The skeleton belonged to a very large male, in size similar to the largest ones from Neumark-Nord 1 (23) and estimated to be around 35 to 40 y old at death, with a head/withers height of 4.20 m (21).

The rescue excavation documented the spatial distribution of 195 complete bones, amongst which 26 flint artifacts were scattered, obtained from 5 to 7 different lithic raw material units (24). The anatomical association of individual body parts of the carcass was still recognizable, but the position of the bones indicates that body parts were disarticulated. For instance, the spatial distribution (Fig. 2) shows two distinct concentrations of disarticulated metapodials within the excavated area, in the southern and northern half, respectively, each within a limited area of one square meter. The skull was removed from its anatomical association and deposited ventrally. Underneath the skull was the left tusk, which had been detached from the alveolus. Parts of the spine and thorax, the left lower leg, and both *Femora* were also disarticulated. About half of the ribs of the left rib cage, on which the entire carcass came to rest, were not present anymore.

The Gröbern elephant is now part of an exhibition installation at the Landesmuseum für Vorgeschichte in Halle (Germany). About half of the excavated bone material was used for this exhibit and hence no longer accessible for study purposes. However, a major part of the bones is kept available for research in the reserves of the Landesdenkmalamt Sachsen-Anhalt in Halle and formed the basis for our studies (*SI Appendix*, Table S1).

## Taubach.

In the early 20th century, Taubach, near Weimar (Germany), had become one of the most important palaeontological and archaeological sites in Europe, because of the large quantities of fossil remains of a wide range of animals recovered during small-scale quarrying work in the travertine sequences there (25). Its early discovery implies that the material from the site is generally not well provenanced, and already during recovery biased in favor of easily identifiable and paleontologically “interesting” pieces, now widely distributed over European museums. In 1870, the first flint artifacts were found, stimulating intensive studies of the fauna yielding fine-grained sands and silts at the bottom of the travertine sequence, described as locally saturated with fossils (8), and containing patches of heated bone and fire-reddened pieces of travertine, compacted with charcoal pieces into localized concentrations of charred materials. The excellent preservation of mollusks and bones in the travertine–sand matrix points to deposition in a low-energy environment on the shores of a shallow lake, with frequent fluctuations in the water level. The bulk of the material can be dated to the early part of the Last Interglacial, probably from PAZ III or later (8).

There seem to have been remarkable concentrations of elephant remains, with an MNI of 40 in February 1891 increasing to 50 in the autumn of that same year (8).

For this project, we studied the elephant assemblage stored at the Senckenberg Forschungsstation für Quartärpaläontologie (Weimar, Germany). This collection, though small in number (*SI Appendix*, Table S2), constitutes the largest sample of Taubach fossils known and is the one with the clearest provenance data (8).

## Results

### Gröbern.

**Fig. 3.**
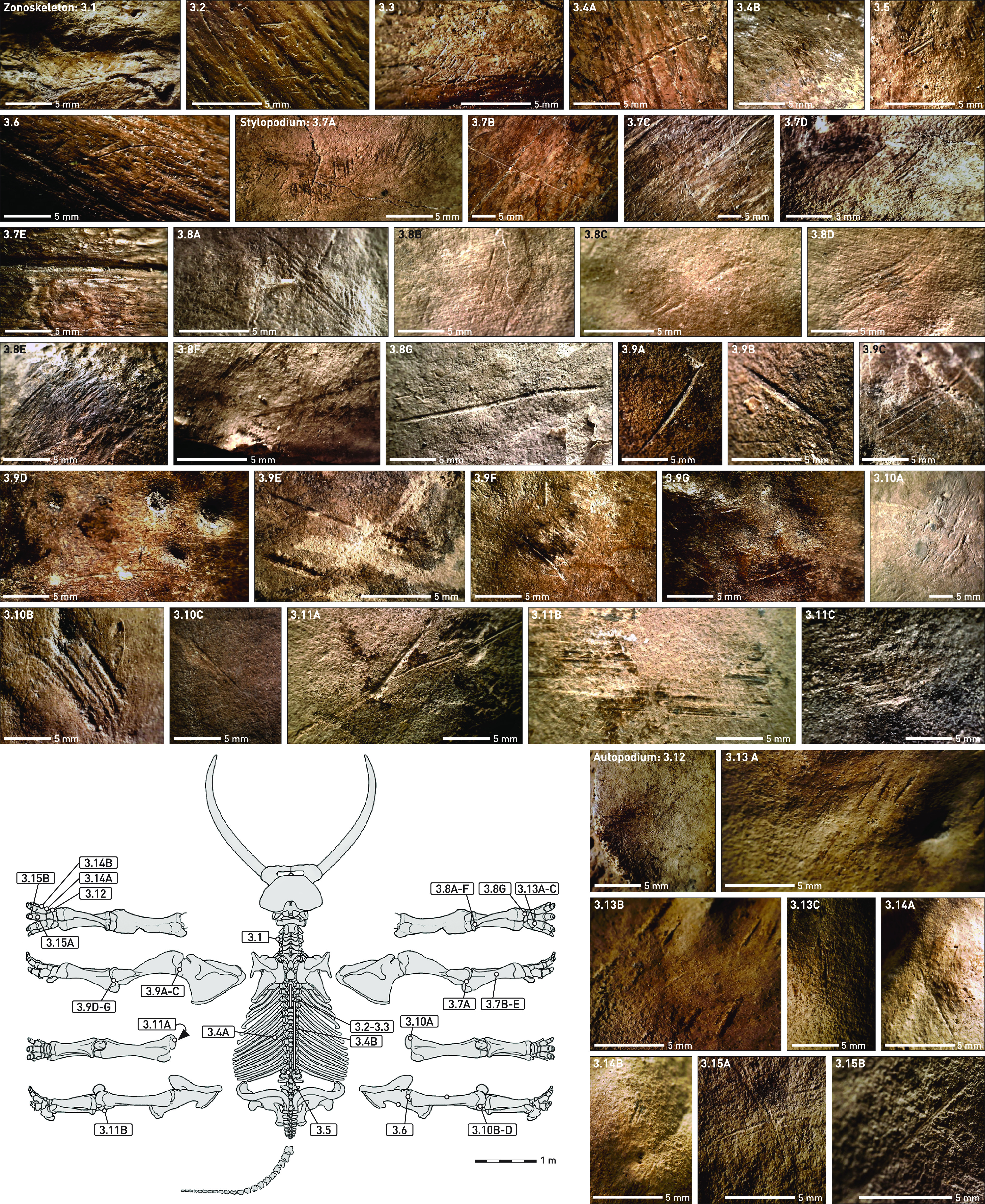
Cut marks on the *P. antiquus* skeleton from Gröbern. The position of cut marks on the elephant skeleton is indicated by numbers. For detailed descriptions see text.

The studied part of the Gröbern carcass displays abundant cut marks (Fig. 3 and *SI Appendix*, Table S1), a record that fits in the “extended utilization” pattern described by Haynes (14). The cut marks are distributed over the left and right side of the zonoskeleton, stylo-, zeugo-, and autopodium. They indicate the exploitation of all body parts of the entire carcass (*SI Appendix*, Table S3). The skull has only been preserved in fragments. Our sample contained only one cervical vertebra fragment, which was cut-marked; hence, the handling of the skull during butchering cannot be inferred through cut-mark evidence. However, the spatial distribution data show that the animal’s skull was inverted, which implies detachment of the head from the trunk, as well-documented by cut marks on the occipital bone of *P. antiquus* from Neumark-Nord. By reaching through the occipital opening of the detached head, the brain of the animal can be removed. Through the spatial distribution data, it is also clear that the tusks of the animal were removed from the jaw.

The cut mark evidence shows that the backstrap muscle/tenderloin on both sides of the body was removed [cuts on the *Processus spinosus* of thoracic vertebrae (Fig. 3.2 and 3.3)] and the ribcage was dissected from the vertebrae [cuts on the *Collum costae* of the first lumbar vertebra (Fig. 3.5) and a *Caput costae* (Fig. 3.4*B*)]. Skin, fat, and connective tissue were removed from the rib cage [cuts on a rib midshaft (Fig. 3.4*A*)].

The carcass was eviscerated (cuts on the ventral surface of the *Os ischium* of the *Pelvis*). Both left and right hindlegs were separated from the *Pelvis* [cuts on the *Acetabulum* of the right *Pelvis* (Fig. 3.6) and on the *Caput ossis femoris* of both right and left *Femora* (Fig. 3.10 *A–D* and 3.11*B*)] and the right hindleg was defleshed (cuts on *Corpus ossis femoris* of the right *Femur*) and disarticulated [cuts on *Condylus lateralis* and *medialis* of the right *Femur* (Fig. 3.10 *B–D*)].

Bones from the left [cut marks on the proximal (Fig. 3.9 *A–C*) and distal (Fig. 3.9 *D–G*) joints of the left *Humerus*] and right forelegs [cuts on the proximal *Ulna* (Fig. 3.7*A*)] and proximal (Fig. 3.8*G*) and distal *Radius* (Fig. 3.8 *A–F*) were equally disarticulated and defleshed [cuts on the cranial/lateral midshaft of the right *Ulna* (Fig. 3.7 *B-E*)].

Removal of the fatty foot cushion in the left and right forefoot left numerous traces on the bones of the autopodium (Fig. 3.12–3.15).

In sum, the data on anthropogenic modifications of the Gröbern elephant clearly demonstrate that the complete animal was butchered in a similar manner as described for the elephants at Neumark-Nord [compare (10) figure S1 to Fig. 3]. As in the case of the large Neumark-Nord 1 elephant assemblage, none of the animal’s bones was broken, in line with the inferred lack of free marrow in the limb bones of *P. antiquus* (26). Age and sex (an older adult male) also align very well with the Neumark-Nord 1 data.

In addition to anthropogenic modifications, we also observed some carnivore traces. The tail of the Gröbern elephant was gnawed, as evidenced by a total of 5 caudal vertebrae with tooth marks.

### Taubach.

**Fig. 4.**
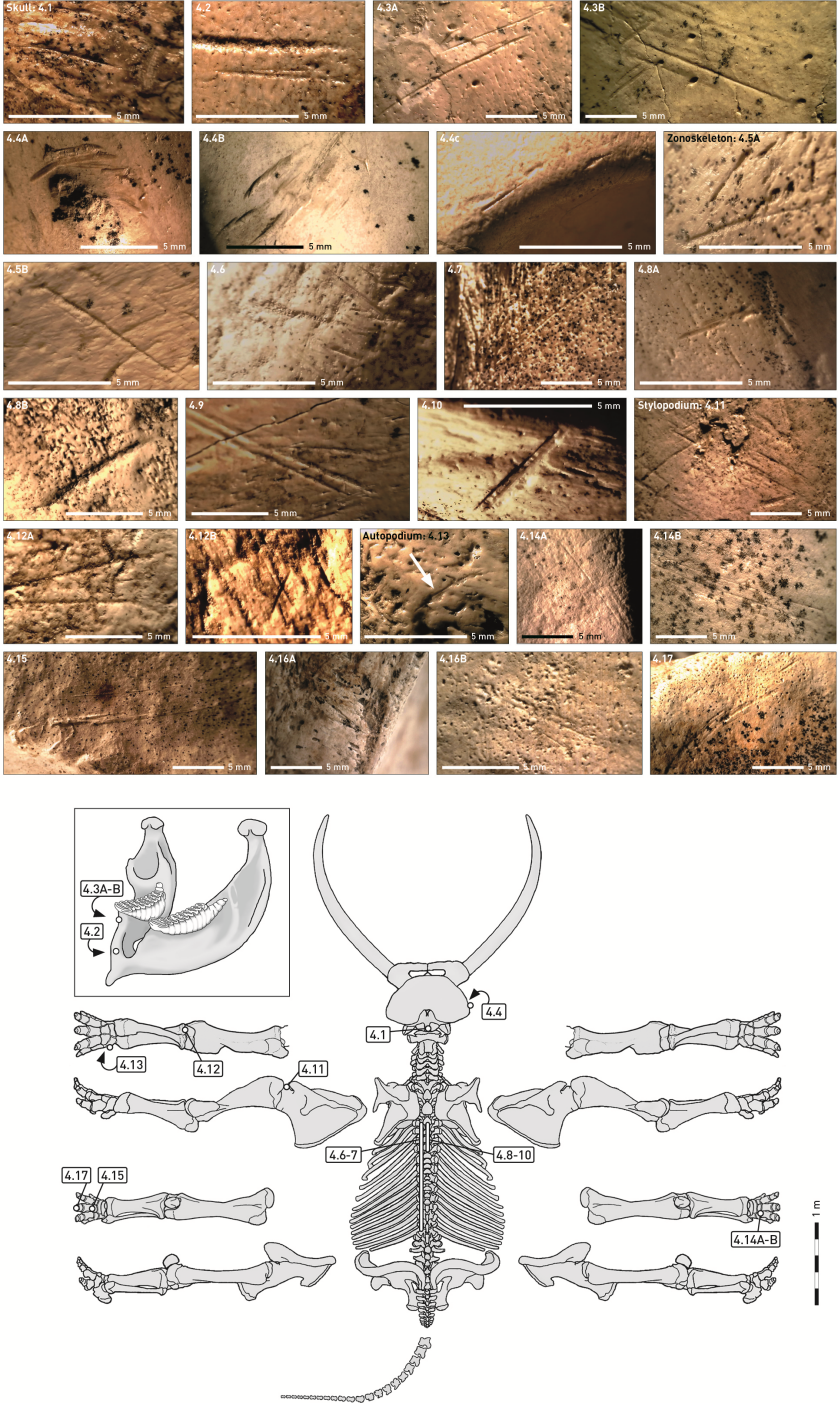
Cut marks on *P. antiquus* remains from Taubach. The position of cut marks on the skeletons is indicated by numbers. For detailed descriptions see text.

Given the way the Taubach material was retrieved and curated, the behavioral resolution is significantly lower here than at Gröbern. The assemblage studied (*SI Appendix*, Table S2) is a small fraction of the large amount of *P. antiquus* material originally recovered from the travertine sequence. Nevertheless, our study yielded relevant information. Of the 166 identified elements, 92 consist of isolated molars. The dominance of these molars (MNI 40) results from selective collecting practices. Beyond the isolated dental remains, the studied sample included 74 cranial elements and bones and bone fragments, which only in a few cases represent more than one (adult) individual. From among these items, 17 display cut marks (Fig. 4), a small number that nevertheless allows us to confidently infer butchering practices comparable to Neumark-Nord and Gröbern:

Cut marks on the inner side of a skull fragment (Fig. 4.1) indicate the removal of the brain. The mandibles were defleshed, and the palate and tongue were removed [cut marks on mandibles and a molar (Fig. 4.2–4.4)].

The trunk was defleshed [cut marks on ribs and of thoracic vertebrae (Fig. 4.7–4.10)], and the thorax was broken open [cut mark on a rib (Fig. 4.6)]. Evidence of hindquarters deboning [cut marks on the *Os ilium* of a *Pelvis* (Fig. 4.5)] and disarticulation [cut marks on the *Collum scapulae* of a left *Scapula* (Fig. 4.11)] and disarticulation of the foreleg [cut marks on a proximal *Ulna* (Fig. 4.12)] is provided.

As in Neumark-Nord and Gröbern, several cut marks (Fig. 4.13–4.17) indicate extraction of the energy-rich fat reserve in the fore and hind feet.

Although only a small part of the originally recovered material could be studied, the cut marks show that the butchering process involved the left and right parts of carcasses and all body parts of the elephant skeleton (*SI Appendix*, Table S2 and Fig. S2).

In addition to the butchering traces left by humans, carnivore modification was also observed. Gnawing marks of large carnivores were found on the proximal ends of the spinae of two vertebrae as well as on the unfused distal epiphysis of a *Femur* and on the distal end of an *Ulna*, which was characterized by a human cut mark in the proximal end. Mesocarnivores left punctures and furrows on the fragment of an occipital bone.

Compared to Neumark-Nord and Gröbern, the elephants in the Taubach collection are relatively small, but ontogenetic bone development points to adult individuals. For 86 elephant molars in our study, we were able to assign the age at death based on tooth formation, eruption, and wear (*SI Appendix*, Text). Based on these data, an MNI of 40 elephants can be established, comprising 25% juvenile, 65% adults, and 10% old individuals. Amongst the adult individuals, 15.4% were younger than 24 y, 38.5% between 25 and 36 y, and 46.2% between 37 and 48 y at death. The dominance of adult individuals, including older ones, strongly resembles the mortality profile from Neumark-Nord, which can be characterized as a prime-age oriented one (*SI Appendix*, Text). However, in contrast to Neumark-Nord and Gröbern, the fragmentary state of preservation of the Taubach bones inhibits unambiguous sex determination.

### In sum.

The cut mark distribution pattern from Gröbern and, to a lesser degree, that from Taubach as well, resemble the pattern established for the Neumark-Nord material. At all three sites, fresh carcasses were extendedly utilized [*sensu* Haynes and Krasinksi (14)], with the removal of almost all edible tissue, disarticulation of joints, and attention to foot bones to get access to the fat-rich foot cushions, creating comparable cut mark distribution patterns (*SI Appendix*, Tables S3 and S4). At Gröbern, all body parts were butchered, from both the left and right side of the adult male individual, as was also the case at Taubach. As at Neumark-Nord, the scarcity of carnivore traces at both sites can be seen as an argument against any postmortem interval before Neanderthal butchering, while the scarcity of postbutchery scavenger tooth marking suggests that Neanderthals stayed some time at the carcass site(s) to process the animal, keeping scavengers away. At Gröbern, carnivore interference may have been limited to a wolf-sized carnivore playing around with (part of) the elephant’s tail.

## Discussion

During minimally 2,000 y in the early part of the Last Interglacial, Neanderthals routinely targeted *P. antiquus* individuals on the northern European plain, as shown by the evidence from Neumark-Nord, Gröbern, and Taubach, rare exposures of Last Interglacial sediments, and the only sites of this time slice in Europe that permit this type of elephant-focused research. For two of these sites, Gröbern and Taubach, we do not have data of sufficient quality to be certain about the way these animals were procured, unlike the hunting evidence from Neumark-Nord. The material available however minimally indicates extended utilization of the elephants similar to Neumark-Nord, with its solid evidence for elephant hunting (18).

Beyond these three sites with good cut mark evidence discussed here, there is also the well-known Lehringen “spear” site, ~200 km northwest of Neumark-Nord, situated in the above-mentioned broad strip of land between the Saalian and the Weichselian ice limits (Fig. 1). There, a lance made out of yew and flint artifacts were associated with the skeletal remains of a large, ~40-y-old male elephant (27–29), also dating to the earlier part of the Last Interglacial PAZIII/IVb (30). The two dozen flint artifacts recovered are strikingly similar to the ones from Gröbern (31), and as at Gröbern, hail from minimally 4 different raw material units (28). No cut marks have been identified on the few (29) skeletal remains that survived the chaotic recovery of the Lehringen finds in 1948. It is very probable that originally a complete skeleton was present (27), with many more flint artifacts (28), most of which had already been quarried away when archaeologists arrived on the scene of discovery; many of the remaining bones ended up in private collections (32).

The Neumark-Nord data show that elephant hunting and exploitation was part of the Neanderthal subsistence repertoire and widespread on the North European plain, already at the very beginning of the Last Interglacial. The knowledge, tools, and skills for successful exploitation of these animals must have been acquired in earlier periods, while targeting other medium-sized and larger mammals, including *P. antiquus* as well as other proboscideans, e.g., of the mammoth lineage during MIS 6 and before, but the record does not allow us to be more specific.

There exists, for example, some tantalizing evidence for *P. antiquus* exploitation from the Lower Travertine deposits at Weimar-Ehringsdorf, dated to MIS 7. This location yielded a rich faunal assemblage, including numerous *P. antiquus* remains, as well as stone artifacts and various Neanderthal fossils (33, 34). In the mid-1930s, travertine exploitation uncovered a complete elephant skeleton, scattered over an area of about 30 m^2^, and associated with lithic artifacts (35). The age profile of the Lower Travertine elephants, based on molars, suggests that 40% of the animals were 20 to 50 y of age at death, while 20% were older (35, 36): a high percentage of adult individuals, similar to Neumark-Nord.

Ehringsdorf is one of the sites featured in recent reviews of the archaeological record of human-elephant interactions in the Pleistocene (37, 38). These studies note an increase in the number of sites comprising proboscidean remains associated with lithics, especially from the middle part of the Middle Pleistocene, ~450 ka ago, onward, in western Eurasia. In Haynes’s words: “…Lower Palaeolithic/Early Stone Age hominins created far fewer proboscidean site assemblages than hominins in later Palaeolithic phases, despite the time span being many times longer. Middle Palaeolithic/Middle Stone Age hominins created assemblages at eight times the earlier hominin rate. Upper Palaeolithic/Later Stone Age hominins created site assemblages at >90 times the rate of Lower Palaeolithic hominins”. Unambiguous cut marks, however, are few and far between in the Lower and Middle Pleistocene record, and if any occur, they do so in very small numbers only (38).

All in all, the quality of the records at pre-Last Interglacial sites differs substantially from that at the Last Interglacial sites discussed here. Neumark-Nord, a palaeolandscape exposed over an area of about 25 ha, has a very rich cut mark record as well as good age and sex profile data; the small (40 m^2^) Gröbern excavation has excellent cut mark evidence and a primary association with stone tools, as does, to some degree, the Taubach material, obtained during quarrying of a 3.5 ha large travertine area. The earlier (MIS 7 and earlier) sites do contain evidence for the co-occurrence of stone tools with elephant remains but thus far they lack a clear-cut mark record of anthropogenic interference.

Based on Churchill’s (39) ethnographic data, one can infer that hunting of large elephants, no matter how dangerous (17), may have required little technological sophistication, with hunting strategies mostly aimed at limiting the mobility of prey, e.g., by digging pits or driving animals into mud traps that may have been present in abundance around the sites discussed here. However, hunting and subsequent processing did require cooperation and an investment of time. If meat and fat were prepared for storage, this would have added to the time investment. Lupo and Schmitt (40), for instance, calculated 745 person-hours for butchery, drying, and smoking of African forest elephants, with a weight of 2 to 4 metric tons only. We previously (10) estimated that skinning, stripping meat from bones, and drying or smoking the meat from an adult male *P. antiquus* could be done in 3 to 5 d, if 25 individuals were involved. Regardless of the value of such estimates, the amounts of food generated by extended utilization of these large animals—to repeat: a 10 metric tons individual, for example, would have easily yielded more than 2,500 daily portions of 4,000 Kcal for adult Neanderthals (10).

The swift processing and extended utilization of these elephants could be interpreted in two ways (10, 18). It could indicate that Neanderthals had cultural means of storing vast masses of fat and meat, e.g., by drying and smoking, and that small groups camped around such stored foods for months. The presence of anthropogenic fire is well-attested at both Neumark-Nord (e.g., refs. 41–43) and at Taubach (44, 45), and while its role in food preservation may seem very plausible, it is archaeologically simply not demonstrable. The same applies to fermentation of meat in caches, recently highlighted as part of the range of hominin food preservation and storage (e.g., ref. 46). Given the lake environments of the sites, storage in water, or “pond storage”, is also a possibility, as it is a “… relatively low-tech, yet safe way of caching meat”, usable in a wide range of environments and through much of the year (ref. 46, p. 56). Again, plausible, but not archaeologically visible.

The repetitive availability of vast amounts of animal tissue mentioned above shows that the potential for large-scale communal subsistence activities was present for Last Interglacial Neanderthals of the northern European plain. Hence, an alternative explanation is that (some of) these elephant kills reflect such short-term larger gatherings. Such an interpretation would raise questions about both the local group size of Neanderthals—generally thought to be much lower than the modern human-gatherer “average” number of 25—and to some degree also about their presumed highly mobile lifestyles (47). The literature routinely emphasizes their small local group size, primarily in the 11 to 16 range (47). Such estimates are hardly based on archaeological data though, limited to the interpretation of spatial patterns—occupational surface areas—at archaeological sites (48, 49) and on some footprint data (50, 51). A recent high-resolution genetic study also suggests a small local group size for Neanderthals from 51 to 59-ka-old levels at Chagyrskya Cave, at the easternmost edge of the known Neanderthal range (52). With strong evidence for patrilocality, here and at El Sidron, Spain (53), the best fitting scenarios assumed a population size of ~20, with smaller probabilities for larger community sizes (of up to 300 individuals). The authors caution against extrapolating their findings to other areas and periods: The Neanderthals sampled in their study may constitute a snapshot part of a larger social group, in which multiple core units, such as nuclear families, repeatedly coalesced, intermixed, and dispersed. Such fluid local groups could have been embedded in larger-scale regional networks, as suggested by lithic raw material transport distances of more than 100 kms, well-documented from the early Middle Paleolithic of Europe onward (54, 55).

The small numbers routinely quoted for Neanderthal local group sizes are strongly rooted in comparative ethnographic data, with a prominent role for the 1968 “Man the Hunter” conference (56). However, as a caveat against too easily extrapolating ethnographic data into the Pleistocene, Birdsell (57) already stressed that “…where large food resources are concentrated either regionally or seasonally, local groups take on a very different nature,” and display such a wide range of adaptive behavior “…that Pleistocene reconstructions will largely depend upon archeological evidence” (ref. 57, p. 236–237). Furthermore, Singh and Glowacki (58) recently pointed out that the focus on mean local group sizes masks strong temporal variation, manifesting both in seasonal fluctuations and in large, periodic aggregations, documented to amount to hundreds of individuals, also for small-scale mobile foragers like the Arrernte of Australia. In the same vein, recent work by Bird et al. (59) emphasizes that while extant residential and foraging groups are indeed often small, “…there is little evidence that these groups are drawn from small communities nested within small-scale societies.” Most mobile hunter-gatherers live in groups bonded by kinship connections as well as containing many non or distant kin, with residential group membership fluid and supporting large-scale social networks of interaction. Indeed, Migliano et al. (60) suggest that a fluid social structure with multiple levels of clustering in social networks was already characteristic for late Middle Pleistocene hunter-gatherers.

The elephant evidence presented here suggests that—at least temporarily—local group sizes *may* have exceeded the small numbers currently dominating reconstructions of the Neanderthal niche. Pleistocene foragers living in environments with rich, dense, and predictable resources, to some degree even cocreated by their landscape modification activities (43), may have coalesced into short-term larger groups. The repetitive food bonanzas documented on the north European plain, seen in the context of the rich Last Interglacial environments filled with other, less dangerous large mammals that at Neumark-Nord were hunted all year round and in large numbers (61), could have provided occasions to gather in larger groups or even have been carried out explicitly in the contexts of such short-term aggregations.

Like the storage-hypothesis, such a larger group size-explanation is difficult if not impossible to test with archaeological data though: As a thought exercise envisaging a “hamlet” of dozens of temporarily aggregating Neanderthals on the higher lake shores overlooking the Neumark-Nord water bodies is one thing, excavating scientific evidence in support of such a scenario is nearly impossible: As almost always with well-preserved Palaeolithic open-air sites, we only have information on the “wet” side of these interglacial adaptations here. In support of a temporary presence of large groups, one could invoke the fact that the lake shore area with the butchered Neumark-Nord elephants contained large high-density distributions of lithics, fragmented bones, and charcoal, documented in rescue excavations at Neumark-Nord 1 (*SI Appendix*, Fig. S3). At Neumark-Nord 2, we documented a small part of this vast distribution in a long-term excavation (2004–2008): Find-level 2/2B, dating to the period in which most elephants were butchered, yielded about 20,000 flint artifacts, more than 118,000 well-preserved faunal remains, heated lithics and charcoal, clustered within an excavated area of 491 m^2^ (61). In all probability, this Neumark-Nord 2/2 and the Neumark-Nord 1 lake-shore scatters constituted the margins of settlements higher up on the shores, eroded during the following glacial. The Taubach elephants also were surrounded by high-density distributions of archaeological materials, their sedimentary matrix described as containing flint artifacts, patches of heated bone, and fire-reddened pieces of travertine, compacted with charcoal pieces into localized concentrations of charred materials (8).

However, despite the exceptionally high temporal resolution of the Neumark-Nord record (43, 62), it is not possible to establish whether these high-density scatters reflect repeated activities of large groups or the accumulated traces of many smaller ones. This ambiguity results from the palimpsest character of the archaeological record, which even at high-resolution sites sets limits to fine-grained “snapshot” readings of our data and hence limits what we can know about past behavior.

What we do know through the elephant exploitation data presented here is that on the North European plain, Last Interglacial foragers were capable of dealing with large amounts of meat and fat in prey processing activities, whether it was through cultural ways of storage, through (short-term) coalescence of large groups, or a combination of both. This evidence provides a unique frame of reference for the interpretation of large accumulations of cut-marked and broken faunal remains at other sites (10): It points to the possibility that accumulations of faunal remains at Neanderthal sites are not necessarily palimpsests of many small-scale hunting and processing events. Like the elephant evidence, such accumulations may indicate the occurrence of larger-scale subsistence activities already during the Middle Paleolithic, facilitated through cultural ways of food storage and/or spatiotemporal variations (fission/fusion) in local hunter-gatherer group size (63).

## Materials and Methods

### Assemblage Composition.

The sample analyzed from Gröbern comprised 58 skeletal elements of *P. antiquus*. Descriptions by Erfurt and Mania (21) served as a point of departure for this study. The skeletal material examined represents that part of the Gröbern elephant carcass that remained in storage for study purposes and was not part of the exhibit in the Landesmuseum für Vor- und Frühgeschichte in Halle (Germany). All available skeletal elements were examined for bone surface modifications (*SI Appendix*, Table S1). The Gröbern elephant remains are kept at the Landesamt für Archäologie Sachsen-Anhalt (Halle, Germany).

The Taubach material studied consisted of 166 skeletal elements of *P. antiquus*. This material (*SI Appendix*, Table S2) represents the largest coherent sample of finds from this site, kept at the Senckenberg Forschungsstation für Quartärpaläontologie (Weimar, Germany).

### Methods.

#### Taxonomic, age, and sex determination.

The *P. antiquus* carcass discovered at Gröbern was paleontologically studied and described by Erfurt and Mania (21). These studies yielded data on age at death, sex, and head/withers height.

The fossil remains from Taubach have been described paleontologically in a special volume dedicated to the site and its geological setting (64). Within this project, the molars of *P. antiquus* were studied by Günther (65), who was able to distinguish 5 female and 4 male specimens by tooth size. Based on Günther’s study of the molars, age at death was calculated following the concept of “African (elephant) equivalent years (AEY)” established by ref. 66 and constantly refined since (11, 67, 68). See also *SI Appendix*, Text.

#### Number of identified specimen per taxon (NISP) and minimum number of elements (MNE).

For the taxonomic determination of bones and bone fragments in our study we used refs. 69–72, as well as complete bones from *P. antiquus* deriving from the Neumark-Nord 1 sample (10). Minimum Numbers of Elements were calculated from the NISP using ref. 73.

#### Minimum number of individuals (MNI).

For the Taubach complex, the MNI was derived from clustering of individual ontogenetic tooth development and wear at age of death, following criteria originally established by Laws (66) (see *SI Appendix*, Text for more information).

#### Bone surface modifications.

Bone surface modifications were studied using hand-held lenses with a magnification of up to 10×, a Dino-Lite PRO digital microscope with a magnification of up to 200× and a Leica reflected-light microscope with a magnification of up to 32×. For each bone or bone fragment, the location of the observed traces on the bone was photographed with a NIKON camera, while close-ups of the bone surface modifications were produced with the Dino-Lite PRO equipment. Traces caused by biotic and abiotic agents were identified using the Taphonomic collection of the Archaeological Research Centre and Museum for Human Behavioural Evolution, MONREPOS, and diagnostic criteria published by ref. 74.

## Supplementary Material

Appendix 01 (PDF)

## Data Availability

All study data are included in the article and/or *SI Appendix*.
