# Supplementary material for: Widespread evidence for elephant exploitation by Last Interglacial Neanderthals on the North European plain
Source: Proc Natl Acad Sci U S A. 2023 Dec 4;120(50):e2309427120. doi: 10.1073/pnas.2309427120 (PMC10723128; doi:10.1073/pnas.2309427120)
Supplement: Supplementary file 1 — Appendix 01 (PDF) [file pnas.2309427120.sapp.pdf]

PNAS

Supporting Information for

Widespread evidence for elephant exploitation by Last Interglacial Neanderthals on the North European plain

Sabine Gaudzinski-Windheuser, Lutz Kindler, Wil Roebroeks

Corresponding author: Wil Roebroeks

Email: w.roebroeks@arch.leidenuniv.nl

This PDF file includes:

Supporting text

Figures S1 to S3

Tables S1 to S4

Supporting Text – Elephant age determination, Minimum Number of Individuals (MNI) and mortality profile of Taubach compared to Neumark-Nord data

Most faunal remains from Taubach were retrieved within a relatively short period during the late nineteenth century. In her study of the rhinoceros assemblage from the site (MNI 76), Bratlund (8) mentions remarkable concentrations of elephant remains, with an MNI of 40 in February 1891 increasing to 50 in the autumn of that same year. Three decades later, Soergel (36) mentions an MNI of 64 straight-tusked elephants, based on dental remains. He concluded that a quarter of the Taubach elephants were less than 6 years of age at death; 28.8 % were between 6-20 years; a comparable percentage 20-50 years old; and 16.7% were more than 50 years old. The embedding of the bones of the elephants (and other animals) occurred in parallel with the fossil-bearing sands, from which Soergel concluded that the animals did not die of a catastrophic event: in his view, these were prey animals, hunted repeatedly close to a water body near the river Ilm, with a focus on young individuals.

Günther (65) obtained similar results in a detailed analysis of 112 elephant tooth remains from Taubach in the Weimar collection (former Institut für Quartärpaläntologie, today Senckenberg Forschungsstation für Quartärpaläontologie). He could assign 99 teeth to a certain molar type (dM1 - dM3, M1-M3) and position in the upper or lower jaw. From these 99, he described 79 molars in detail and noticed the remarkably high number of shed molars in the assemblage (65). His age profile of the Taubach elephants is not based on age at death per individual, but on the number of represented molar types as well and their respective year of eruption and ejection (65), and also included the teeth that were shed during the life time of an individual (65). Thus, the age structure established by Günther does not represent a mortality profile. In addition, the six molars of an elephant jaw are replaced successively during the lifetime and "tooth life" increases continuously from the first milk tooth (dm1 = 2 years) to the last permanent molar (M6 = 38 years) (see (11)). Without further information, the likelihood of two isolated M3 deriving from two individuals is much higher than in other molars with shorter "tooth life". Thus, the age structure Günther established (65) could be biased towards younger age classes.

Presently the Taubach material consists in total of 114 molars, either isolated or still attached to the jaw, or as fragmentary lamellae, of which 59 were already presented in detail by Günther. Omitting the shed molars, age at death for each molar was established using the concept of „African (elephant) equivalent years (AEY)" established by Laws (66) (see also (11, 68)), which describes the intervals of eruption, the number of teeth in the jaw, the progression of tooth wear and shedding as well as the successive replacement from the first milk molars to the last permanent molar. Combining the AEY per molar, the molar type and the quadrant of the jaw the molar originates from, as well as the sex of the individuals identified by Günther (65), the teeth represent an MNI of 40.

Following the age subdivision in five intervals each representing ~20% of the maximum expected lifetime in elephants (11, 75), the Taubach assemblage is structured as follows: 0-12 AEY = 10 individuals, 13-24 AEY = 4 individuals, 25-36 AEY = 10 individuals, 37-48 AEY = 12 individuals, >49 AEY = 4 individuals. Supplementary Text Figure 1 displays the Taubach age structure compared to

the one obtained at Neumark-Nord (15, 76) (see also (10)) . In order to distinguish and characterize mortality in fossil assemblages, Discamps and Costamagno (77) provide mathematically derived zonings of juvenile, prime adult and old adult age classes, that can be plotted in relative frequencies in a ternary diagram. The boundaries between different zones of mortality are species-specific, taking the life histories, ontogenies and ethologies of different taxa in account. Subsequently, Haynes (78) adopted this scheme to analyze mammoth age profiles in the fossil record. He provides some arguments to define the juvenile age cohort, which influences the shape and location of the mortality zones in the diagram. Elephants reach sexual maturity at the age of ~12 years, when they still are not fully grown and unexperienced in self-defense. This may justify expanding the juvenile age cohort to 18 years. Individuals between 13 and 18 years were not identified in the Taubach nor in the Neumark-Nord material. The ratio between juveniles, prime and old adult does not change when expanding the juvenile age class from 12 to 18 years. However, as the age range of the juvenile cohort grows and takes a larger share of the potential ecological longevity, the boundaries between the different mortality zones change (see (77)). The results of both scenarios are displayed in Supplementary Text Figure 2. Limiting the juvenile cohort to 12 years, the Taubach mortality profile falls in the outer range of the “juvenile and prime dominant” range, close to the border to the “prime dominant” zone, while Neumark-Nord is located in the “prime dominant” zone. With an expansion of the juvenile cohort to 18 years, the Taubach population too is placed in the ”prime dominant” range, underlining that in both assemblages the majority of individuals died in an older, prime age stage of life.

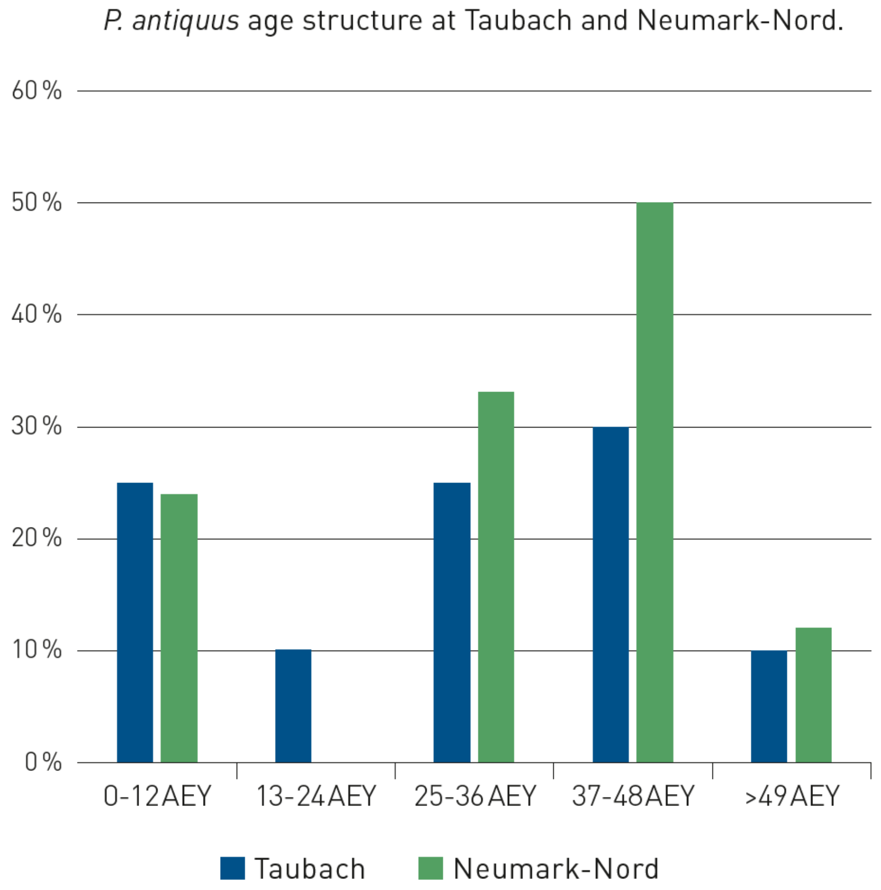

Supporting Text Figure 1: Population structure of *P. antiquus* from Taubach (MNI = 40) and Neumark-Nord (MNI = 24). Age cohorts as established by Haynes (11, 75), 0-12 AEY = juvenile, 13-24 AEY young adults, 25-35 AEY mature adults, 37-48 AEY old adults, and >49 AEY old individuals.

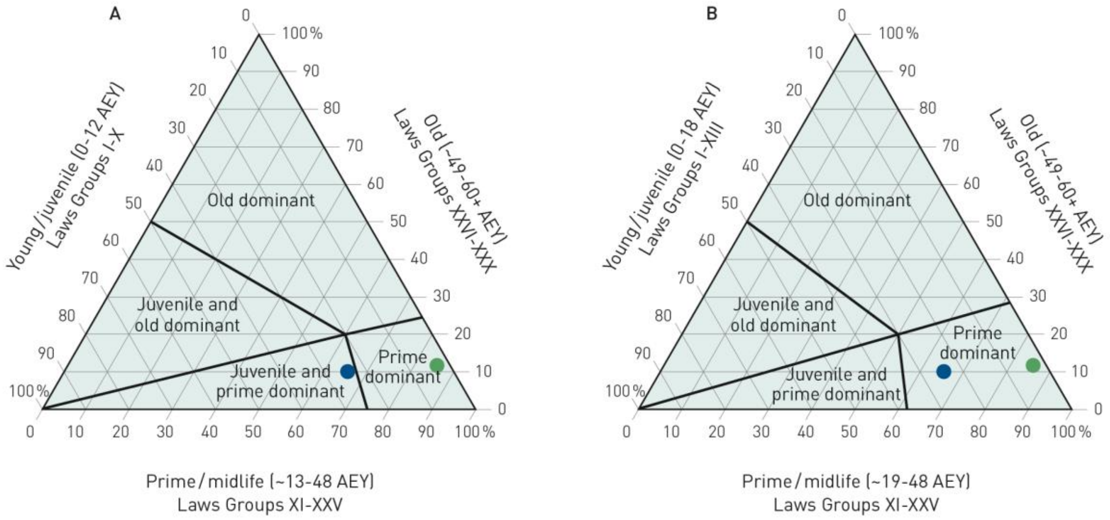

Supporting Text Figure 2: Ternary diagram summarizing population age structures in fossil assemblages (77). Location of the Taubach elephants in blue and of the elephants from Neumark-Nord in orange. A: juvenile age class limited to 12 years and respective zones of mortality. B: juvenile age class expanded to 18 years and respective age class (see (78)).

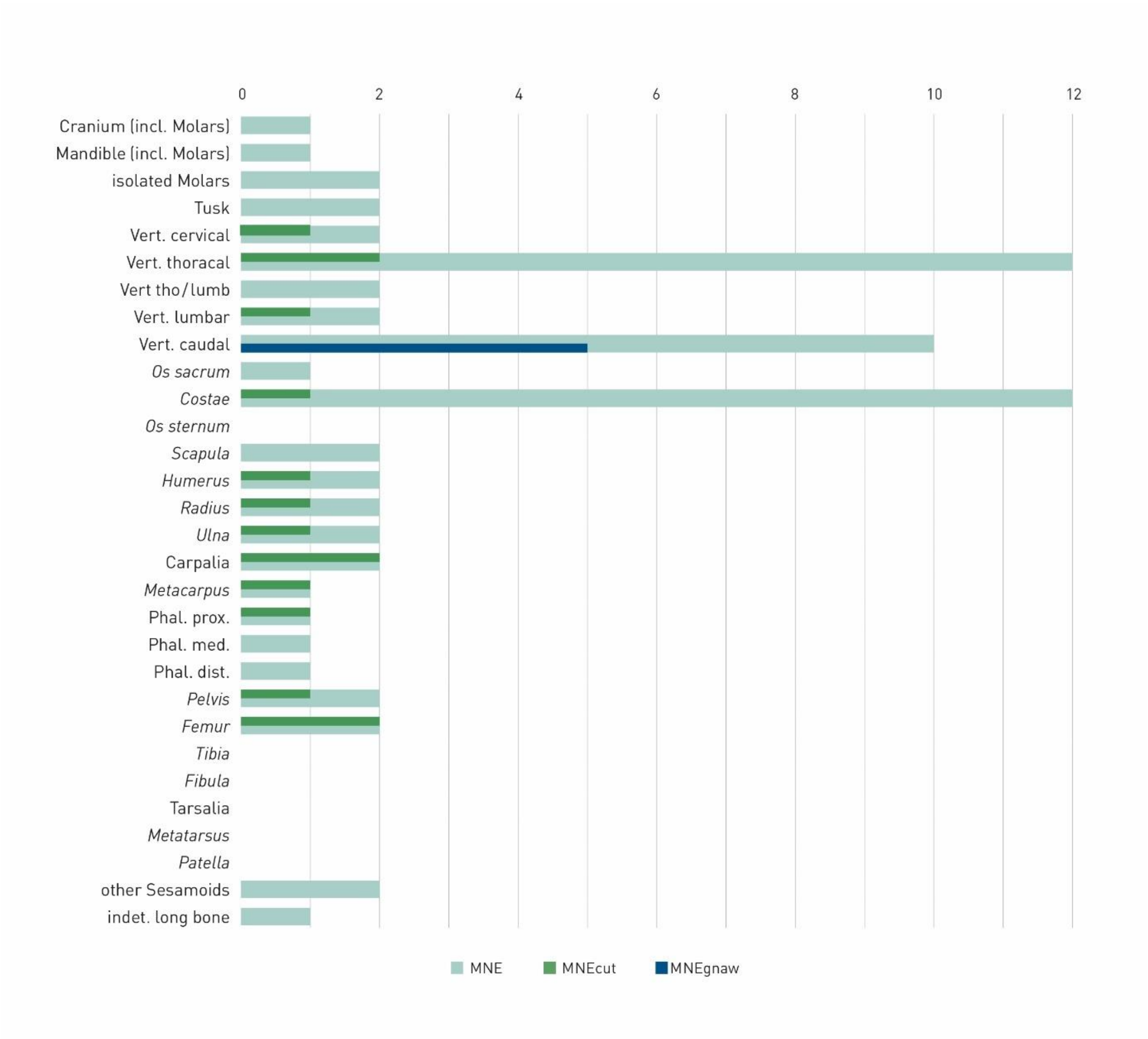

Fig. S1. MNE (minimal number of elements) for bones of *P. antiquus* from Gröbern (light green), MNE of cut marked bones (dark green), and MNE of bones displaying damage induced by large carnivores (blue). For data see Table S1

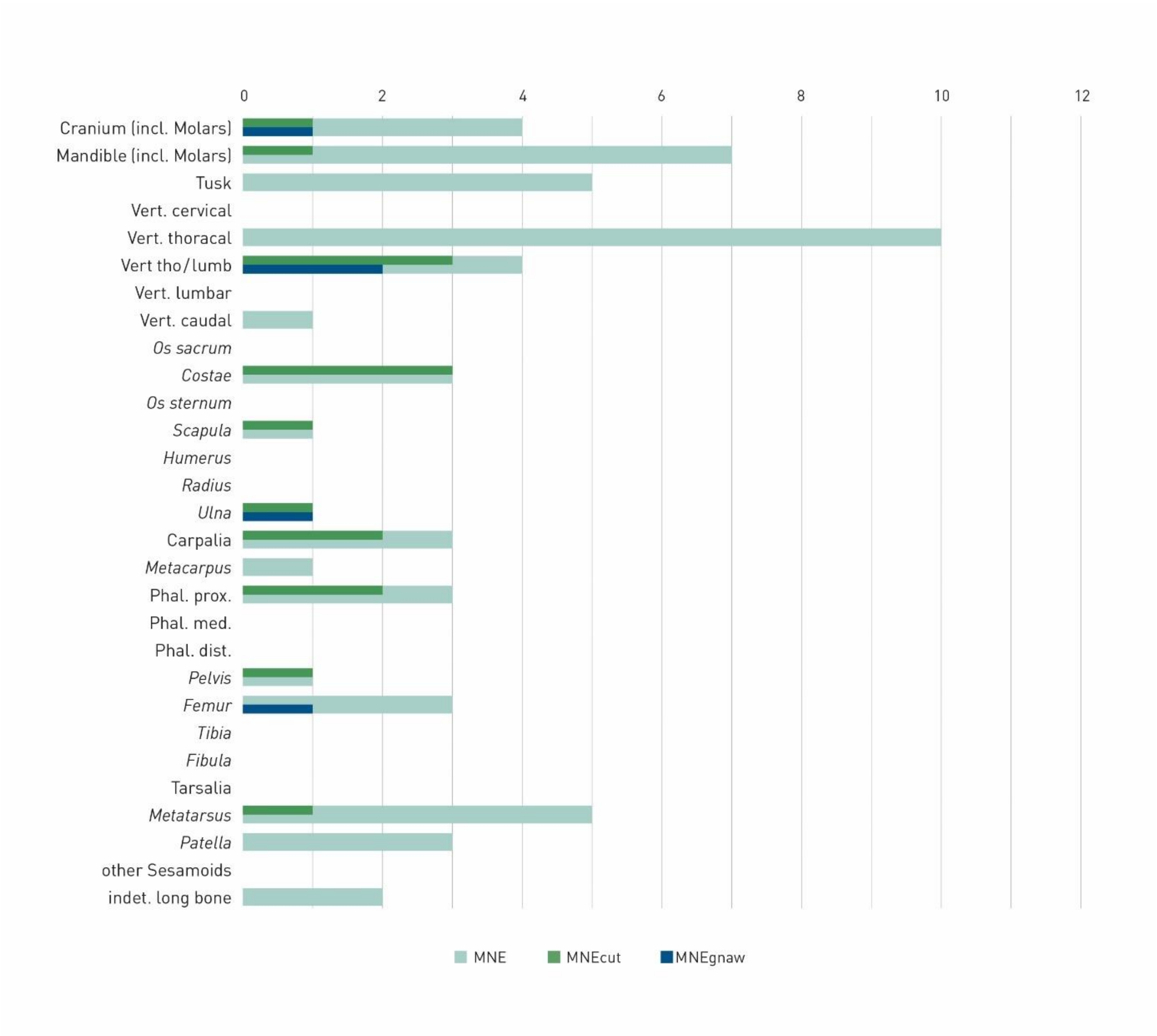

Fig. S2. MNE (minimal number of elements) for bones of *P. antiquus* from Taubach (light green), MNE of cut marked bones (dark green) and MNE of bones displaying damage induced by large carnivores (blue). For data see Table S2

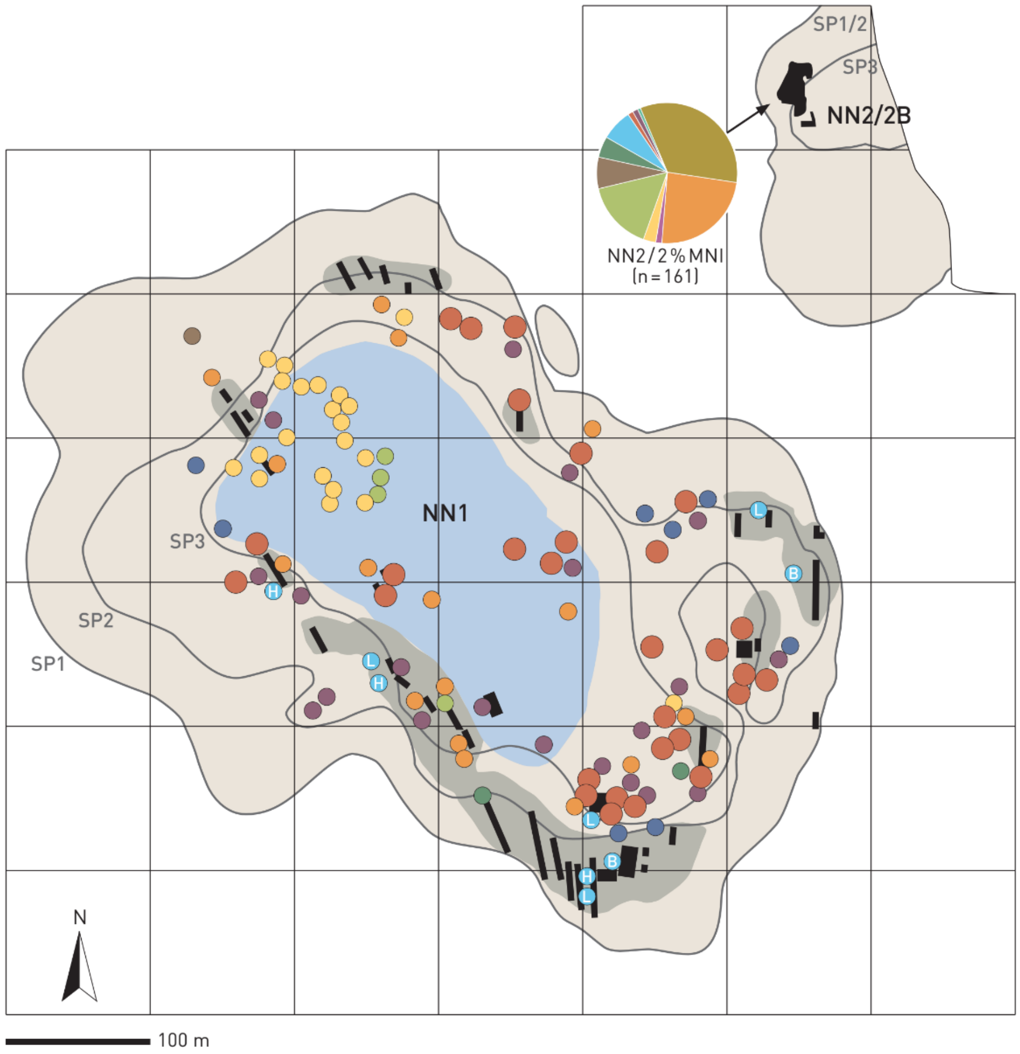

LEGEND

outline of basin NN1 and NN2 in sectional planes (below surface) SP1 = 8 m / SP2 = 16 m / SP3 = 24 m

excavation area

littoral deposits during PAZ IV at NN1 (lower and upper littoral horizon)

extent of water table during PAZ IV at NN1

area of dense archaeological find scatters in the littoral deposits during PAZ IV

Complete and partial skeleton(s):

- Elephant
- Rhino
- Aurochs
- Giant deer
- Red deer
- Fallow deer
- Cervid indet.
- Carnivore / Hyena (H)
- Carnivore / Lion (L)
- Carnivore / Bear (B)
- Carnivore / Canid
- Horse
- Boar
- Roe deer
- Elephant tusk(s)

Fig. S3. Map of the basin Neumark-Nord 1 in the center of the figure, and the small Neumark-Nord 2 basin in the upper right corner, with indicated the locations of the butchered elephants (red dots), and other more or less complete mammal skeletons, the Neumark-Nord 1 archaeological rescue interventions (black rectangles), the high density distributions of flint artifacts and fragmented faunal remains in Neumark-Nord 1 (darker and hatched areas), and the position of the Neumark-Nord 2/2 excavated area (in black), where a small part of this high-density scatter was recorded in detail. The Neumark-Nord 2 pie chart shows the percentage distribution of prey species identified in find level 2/2B (MNI 161). The map displays finds dating to Pollen Assemblage Zone IV of the Last Interglacial only. Note the large size of the landscape sampled in the fieldwork here. SP1, 2 and 3: contour lines of the basins, at respectively 8, 16 and 24 m below the surface. SP 1 indicates the maximum extension of the water body. Based on (61), with data derived from (42), Fig. 28, and (79).

Table S1. The studied sample from Gröbern, listed according to skeletal element. NE skeleton – Number of bones in an elephant carcass, NE - excavated - Number of bones excavated from the elephant carcass at Gröbern, NE - studied - Number of bones studied from the elephant carcass at Gröbern, NE cut marked - Number of cut marked bones.

**Elements**	**NE skeleton**	**NE excavated**	**NE studied**	**NE cut marked**
Cranium	1	1	1	0
Mandible	1	1	1	0
Tusk	2	2	2	0
Vert. cervical	7	6	2	1
Vert. thoracic	19	16	12	2
Vert. lumb	4	4	2	1
Vert. caudal	21	6	6	0
Os sacrum	1	1	1	0
Costae	38	22	12	1
Os sternum	1	1	0	0
Scapula	2	2	2	0
Humerus	2	2	2	1
Radius	2	2	2	1
Ulna	2	2	2	1
Carpalia	16	16	2	2
Metacarpus	10	9	1	1
Phal. prox. man.	10	9	1	1
Phal. med. man.	8	8	1	0
Phal. dist. man.	6	5	1	0
Pelvis	1	1	1	1
Femur	2	2	2	2
Tibia	2	2	0	0
Fibula	2	2	0	0
Tarsalia	14	14	0	0
Metatarsus	10	10	0	0
Phal. prox. pes	8	8	0	0
Phal. med. pes	8	6	0	0
Phal. dist. pes	4	0	0	0
Patella	2	2	0	0
other sesamoids	36	24	2	0
**Sum**	**242**	**186**	**58**	**15**

Table S2. The studied sample from Taubach, listed according to skeletal element. NISP – Number of identified specimen per taxon, MNE – Minimum Number of Elements, NISPcut / MNEcut – Number of identified specimen per taxon with cut marks / Minimum number of cut marked elements, NISPgnaw / MNEgnaw – Number of identified specimen per taxon modified by large carnivores / Minimum number of elements modified by large carnivores.

Elements	NISP	MNE	MNI	NISP cut	MNE cut	NISPgnaw	MNEgnaw
Cranium (incl. Molars)	14	4	4	1	1	1	1
Mandible (incl. Molars)	13	7	7	1	1		
isolated Molars	92	87	17	1	1		
Tusk	5	5	5				
Vert. cervical							
Vert. thoracic	10	10	1				
Vert. tho/lumb	4	4	1	3	3	2	2
Vert. lumbar							
Vert. caudal	1	1	1				
Os sacrum							
Costae	3	3	1	3	3		
Os sternum							
Scapula	1	1	1	1	1		
Humerus							
Radius							
Ulna	1	1	1	1	1	1	1
Carpalia	3	3	1	2	2		
Metacarpus	1	1	1				
Phal. prox.	3	3	1	2	2		
Phal. med.							
Phal. dist.							
Pelvis	1	1	1	1	1		
Femur	3	3	2			1	1
Tibia							
Fibula							
Tarsalia							
Metatarsus	5	5	2	1	1		
Patella	3	3	2				
other sesamoids							
indet. long bone	3	2	1				
indet. fragment							
**Sum**	**166**	**144**	**17**	**17**	**17**	**5**	**5**

Table S3. Cut marks and disarticulation sequences at Gröbern as indicated by location of cut marks and their interpretation. The cut marks are illustrated in Figure 3 in the main text.

Bone	Location of cut marks	Interpretation of cut marks	Figure
ZONOSKELETON			
Vertebra	cervical, *Processus transversus* sin.		Fig. 3.1
Vertebra	thoracal – left lateral, *Processus spinosus* proximal	removal of tenderloin – defleshing the carcass	Fig. 3.2
Vertebra	thoracal – right lateral, *Processus spinosus* proximal	removal of tenderloin – defleshing the carcass	Fig. 3.3
Rib sin.	medial, *Corpus costae*, mid-shaft	removal of skin, fat and connective tissue	Fig. 3.4 A
Rib sin.	*Caput costae*	separating rib from vertebra – disarticulation of the ribcage	Fig. 3.4 B
Vertebra	1st lumbar, *Collum costae* sin.	separating rib from vertebra – disarticulation of the ribcage	Fig. 3.5
Pelvis dex.	*Acetabulum*, on *Area musculi recti femoris*	severing the connection between *Pelvis* and the proximal *Femur* – deboning of the right hindleg	Fig. 3.6
Pelvis dex.	*Os ischium*, ventral	removal of the viscera – the evisceration of the carcass	
STYLOPODIUM & ZEUGOPODIUM			
Ulna dex.	proximal, *Incisura trochlearis* proximal	separating the connection between distal *Humerus* and *Ulna* – severing the connection between right stylo- and zeugopodium	Fig. 3.7 A
Ulna dex.	*Corpus ulnae*, cranial/lateral midshaft	defleshing of the right foreleg	Fig. 3.7 B-E
Radius dex.	proximal on *Fovea capitis radii*	separating the connection between distal *Humerus* and proximal *Radius* – severing the connection between right stylo- and zeugopodium	Fig. 3.8 A-D
Radius dex.	proximal on *Circumferentia articularis*	separating the connection between proximal *Radius* and proximal *Ulna* – disarticulation of the right foreleg	Fig. 3.8 E-F
Radius dex.	distal on *Facies articularis carpea*	separating the connection to *Os carpi ulnare* – severing the connection between right auto- and zeugopodium	Fig. 3.8 G
Humerus sin.	proximal on *Caput humeri*	separating the right *Humerus* from *Scapula* – deboning the left foreleg	Fig. 3.9 A-C
Humerus sin.	distal on *Trochlea humeri*	separating the joint connection to the proximal *Ulna* – disarticulation of the foreleg – separating the connection between left stylo- and zeugopodium	Fig. 3.9 D-G
Femur dex.	proximal on *Caput ossis femoris*	separating the connection between the right proximal *Femur* and the *Acetabulum* – deboning the right hindleg	Fig. 3.10 A
Femur dex.	caudal on *Corpus ossis femori*s, midshaft	defleshing of the right hindleg	
Femur dex.	distal, caudal on *Condylus lateralis* and *Condylus medialis*	separating the connection between distal *Femur* and proximal *Tibia* – severing the connection between right stylo- and zeugopodium	Fig. 3.10 B-D
Femur sin.	proximal on *Caput ossis femoris*	separating the connection between the right proximal *Femur* and the *Acetabulum* – deboning the left hindleg	Fig. 3.11 A
Femur sin.	distal, caudal on *Condylus medialis*	separating the connection between left distal *Femur* and proximal *Tibia* – severing the connection between left stylo- and zeugopodium	Fig. 3.11 B
AUTOPODIUM			
Os carpale II sin.	proximal	severing the connection to the left *Os carpi radiale* – disarticulation of left forefoot	Fig. 3.12
Os carpale IV dex.	distal	severing the connection to the left distal metapodials – disarticulation of the right forefoot	Fig. 3.13 A-C
Os metacarpale I sin.	proximal	severing the connection to the left *Os carpale I* sin. – disarticulation of the left forefoot	Fig. 3.14 A
Os metacarpale I sin.	distal	severing the connection to *Phalange proximale, dig. I* and sesamoid – disarticulation of the left forefoot	Fig. 3.14 B
Phalange proximale, dig. III, man., sin.	proximal	severing the connection to the left distal *Os metacarpus III* – disarticulation of the left forefoot	Fig. 3.15 A
Phalange proximale, dig. III, man., sin.	distal	severing the connection to the left proximal *Phalange media, dig. III* – disarticulation of the left forefoot	Fig. 3.15 B

Table S4. Cut marks and disarticulation sequences at Taubach as indicated by location of cut marks and their interpretation. The cut marks are illustrated by Figure 4 in the main text.

Bone	Location of cut marks	Interpretation of cut marks	Figure
SKULL			
Skull	*Os occipitale*, inside of the skull, next to *Condylus occipitalis* sin.	removal of brain	Fig. 4.1
Mandible dex.	*Corpus mandibulae*, lateral next to *Foramina mentalia lateralia*	defleshing the carcass	Fig. 4.2
Mandible dex.	*Corpus mandibulae*, lateral next to *Pars molaris*	defleshing the carcass	Fig. 4.3 A-B
sup. M3 dex.	dorsal / buccal	removal of palate / tongue	Fig. 4.4 A
sup. M3 dex.	dorsal / laterial	removal of palate	Fig. 4.4 B-C
ZONOSKELETON			
Pelvis indet.	*Os ilium*, lateral	severing the connection between *Pelvis* and proximal *Femur* – deboning the hindleg	Fig. 4.5 A-B
Rib sin.	*Collum costae*, cranial	disarticulation of the ribcage	Fig. 4.6
Rib *indet.*	*Corpus costae*, cranial	removal of skin, fat and connective tissue	Fig. 4.7
thoracic vertebra	*Processus spinosus*, caudal, left lateral, proximal	removal of tenderloin – defleshing the carcass	Fig. 4.8 A
thoracic vertebra	*Processus spinosus*, cranial, right lateral, proximal	removal of tenderloin – defleshing the carcass	Fig. 4.8 B
thoracic vertebra	*Processus spinosus*, right lateral, proximal	removal of tenderloin – defleshing the carcass	Fig. 4.9
thoracic vertebra	*Processus spinosus*, cranial, left lateral, towards distal	removal of tenderloin – defleshing the carcass	Fig. 4.10
Scapula sin.	*Collum scapulae*, medial	detachment of the *Scapula* – dissection of left half of the torso	Fig. 4.11
ZEUGOSKELETON			
Ulna sin.	proximal, cranial, below *Processus anconeus*	separating the connection between distal *Humerus* and *Ulna* severing the connection between the left stylo- and zeugopodium	Fig. 4.12 A-B
AUTOPODIUM			
Os carpale I sin.	distal	severing the connection to left *Os metacarpale I* – disarticulation of the left forefoot	Fig. 4.13
Os carpale IV dex.	medial, proximal	severing the connection to the right *Os carpale III* – disarticulation of the right forefoot	Fig. 4.14 A
Os carpale IV dex.	medial, distal	severing the connection to the right *Os metacarpus III* – disarticulation of the right forefoot	Fig. 4.14 B
Os metatarsale III sin.	medial, proximal	severing the connection to the left *Os metatarsale IV* – disarticulation of the left forefoot	Fig. 4.15
Phalange proximale, dig. III, pes	proximal	severing the connection to the distal *Os metatarsale III* – disarticulation of the hindfoot	Fig. 4.16 A
Phalange proximale, dig. III, pes	distal	severing the connection to the proximal *Phalange media, dig. III*, pes – disarticulation of the hindfoot	Fig. 4.16 B
Phalange proximale sin, dig. III, pes	proximal	severing the connection to the distal *Os metatarsale IV* – disarticulation of the left hindfoot	Fig. 4.17

SI References

1. Gaudzinski-Windheuser S & Niven L (2009) Hominin Subsistence Patterns During the Middle and Late Paleolithic in Northwestern Europe. *The Evolution of Hominin Diets: Integrating Approaches to the Study of Palaeolithic Subsistence*, eds Hublin J-J & Richards MP (Springer, Leipzig), pp 99-111.
2. White M, Pettitt P, & Schreve D (2016) Shoot first, ask questions later: Interpretative narratives of Neanderthal hunting. *Quaternary Science Reviews* 140:1-20.
3. Morin E, Speth J, & Lee-Thorp J (2016) Middle Palaeolithic Diets: A Critical Examination of the Evidence.).
4. Power RC*, et al.* (2018) Dental calculus indicates widespread plant use within the stable Neanderthal dietary niche. *Journal of Human Evolution* 119:27-41.
5. Blasco R & Fernández Peris J (2012) A uniquely broad spectrum diet during the Middle Pleistocene at Bolomor Cave (Valencia, Spain). *Quaternary International* 252:16-31.
6. Hardy BL & Moncel M-H (2011) Neanderthal Use of Fish, Mammals, Birds, Starchy Plants and Wood 125-250,000 Years Ago. *PLoS ONE* 6(8):e23768.
7. Kindler L (2012) *Die Rolle von Raubtieren in der Einnischung und Subsistenz jungpleistozäner Neandertaler. Archäozoologie und Taphonomie der mittelpaläolithischen Fauna aus der Balver Höhle (Westfalen)* (Römisch-Germanisches Zentralmuseum, Mainz).
8. Bratlund B (1999) Taubach Revisited. *Jahrbuch des Römisch-Germanischen Zentralmuseums Mainz* 46:61-174.
9. Auguste P (1993) Acquisition et exploitation du gibier au Paléolithique moyen dans le nord de la France. Perspectives paléo-écoologiques et palethnographiques. *Exploitations des animaux sauvages à travers le temps*, Antibes).
10. Gaudzinski-Windheuser S, Kindler L, MacDonald K, & Roebroeks W (2023) Hunting and processing of straight-tusked elephants 125.000 years ago: Implications for Neanderthal behavior. *Science Advances* 9(5):eadd8186.
11. Haynes G (1991) *Mammoths, mastodonts, and elephants* (Cambridge University Press, Cambridge).
12. Ndlovu M*, et al.* (2023) Age-sex structure of drought-driven African elephant (*Loxodonta africana*) mortality in Hwange National Park, Zimbabwe. *Scientific African* 19:e01459.
13. Mukeka JM, Ogutu JO, Kanga E, Piepho H-P, & Røskaft E (2022) Long-term trends in elephant mortality and their causes in Kenya. *Frontiers in Conservation Science* 3.
14. Haynes G & Krasinski K (2021) Butchering marks on bones of Loxodonta africana (African savanna elephant): Implications for interpreting marks on fossil proboscidean bones. *Journal of Archaeological Science: Reports* 37:102957.
15. Palombo MR, Albayrak E, & Marano F (2010) The straight-tusked Elephants from Neumark Nord, a glance to a lost world. *Elefantenreich-Eine Fossilwelt in Europa.*, ed Meller H (Landesamt für Denkmalpflege und Archäologie Sachsen-Anhalt – Landesmuseum für Vorgeschichte Halle, Saale, Halle), pp 219-247.
16. Larramendi A, Palombo MR, & Marano F (2017) Reconstructing the life appearance of a Pleistocene giant: size, shape, sexual dimorphism and ontogeny of Palaeoloxodon antiquus (Proboscidea: Elephantidae) from Neumark-Nord 1 (Germany). *Boll. Soc. Paleont. Italiana* 56(3):299-317.
17. Lupo KD & Schmitt DN (2023) Reframing Prehistoric Human-Proboscidean Interactions: on the Use and Implications of Ethnohistoric Records for Understanding the Productivity of Hunting Megaherbivores. *Journal of Archaeological Method and Theory*.
18. Starkovich BM (2023) Perception versus reality: Implications of elephant hunting by Neanderthals. *Science Advances* 9(5):eadg6072.
19. Roebroeks W & Speleers B (2002) Last interglacial (Eemian) occupation of the North European plain and adjacent areas. *Le Dernier Interglaciaire et les occupations humaines du*

Paléolithique moyen, eds Tuffreau A & Roebroeks W (CERP/ Université des Sciences et Technologies de Lille, Lille), pp 31-39.
20. Litt T (1990) Stratigraphie und Ökologie des eeminterglazialen Waldelefanten-Schlachtplatzes von Gröbern, Kreis Gräfenhainichen. *Beiträge zur Jagd des mittelpaläolithischen Menschen,* Veröffentlichungen des Landesmuseums für Vorgeschichte in Halle eds Mania D, Thomae M, Litt T, & Weber T (Deutscher Verslag der Wissenschaften, Berlin), pp 193-208.
21. Erfurt J & Mania D (1990) Zur Paläontologie des jungpleistozänen Waldelefanten von Gröbern, Kreis Gräfenhainichen. *Neumark - Gröbern. Beitrage zur Jagd des mittelpaläolitischen Menschen*, eds Mania D, Thomae M, Litt T, & Weber T (Deutscher Verlag der Wissenschaften, Berlin), Vol Veröffentlichungen des Landesmuseums für Vorgeschichte Halle 43.
22. Menke B & Tynni R (1984) Das Eeminterglazial und das Weichselfrühglazial von Rederstall/Dittmarschen und ihre Bedeutung für die mitteleuropäische Jungpleistozängliederung. *Geologisches Jahrbuch* A76:3-120.
23. Marano F & Palombo MR (2011) The straight-tusked elephants from Neumark-Nord 1: the state of the art. *Il Quaternario* 24:147-149.
24. Heussner K-U & Weber T (1990) Das archäologische Inventar - Spezielle Untersuchungen zu den Feuersteinartefakten. *Neumark-Gröbern, Beiträge zur Jagd des mittelpaläolitischen Menschen*, eds Mania D, Thomae D, Litt T, & Weber T (Deutscher Verlag der Wissenschaften, Berlin), pp 225-236.
25. Steiner W & Wiefel H (1977) Zur Geschichte der geologischen Erforschung des Travertins von Taubach bei Weimar. *Quartärpaläontologie* 2:9-81.
26. Haynes G, Krasinski K, & Wojtal P (2021) A Study of Fractured Proboscidean Bones in Recent and Fossil Assemblages. *Journal of Archaeological Method and Theory* 28(3):956-1025.
27. Adam K (1951) Der Waldelefant von Lehringen, eine Jagdbeutes diluvalien Menchen. *Quartär* 5:75-92.
28. Thieme H & Veil S (1985) Neue Untersuchungen zum eemzeitlichen Elefanten-Jagdplatz Lehringen, Ldkr. Verden. *Die Kunde* 36:11-58.
29. Houben C (2003) Die Wirbeltierfauna aus dem letzten Interglazial von Lehringen (Niedersachsen, Deutschland). *Eiszeitalter und Gegenwart* 52:25-39.
30. Selle W (1962) Geologische und vegetationskundliche Untersuchungen an einigen wichtigen Vorkommen des letzten Interglazials in Nordwestdeutschland. *Geologisches Jahrbuch* 79:295-352.
31. Weber T (2000) The Eemian Elephas antiquus finds with Artefacts from Lehringen and Griibern: Are they really Killing Sites. *Anthropologie et Préhistoire* 111:177-185.
32. Deibel-Rosenbrock W (1960) Die Funde von Lehringen (Nach dem Aufzeichnungen ihres Vaters A. Rosenbrock). *Stader Jahrbuch* 1960:3-35.
33. Steiner W (1979) *Der Travertin von Ehringsdorf und Seine Fossilien* (Neue Brehm-Bücherei, Wittenberg).
34. Street M, Terberger T, & Orschiedt J (2006) A critical review of the German Paleolithic hominin record. *Journal of Human Evolution* 51:551-579.
35. Behm-Blancke G (1960) Altsteinzeitlich Rastplätze im Travertingebiet von Taubach, Weimar, Ehringsdorf. *Alt-Thüringen* 4:1-246.
36. Soergel W (1922) *Die Jagd der Vorzeit* (Gustav Fischer, Jena).
37. Konidaris GE & Tourloukis V (2021) Proboscidea-Homo interactions in open-air localities during the Early and Middle Pleistocene of western Eurasia: a palaeontological and archaeological perspective. *Human-elephant interactions: from past to present*, eds Konidaris GE, Barkai R, Tourloukis V, & Harvati K (Tübingen University Press, Tübingen), pp 67-104.
38. Haynes G (2022) Late Quaternary Proboscidean Sites in Africa and Eurasia with Possible or Probable Evidence for Hominin Involvement. *Quaternary Geochronology* 5(8).
39. Churchill SE (1993) Weapon technology, prey size selection, and hunting methods in modern hunter-gatherers: implications for hunting in the Palaeolithic and Mesolithic. *Hunting and*

animal exploitation in the Later Palaeolithic and Mesolithic of Europe, Archaeological papers of the American Anthropological Association, eds Peterkin GL, Bricker HM, & Mellars PA (American Anthropological Association), pp 11-24.
40. Lupo KD & Schmitt DN (2016) When bigger is not better: The economics of hunting megafauna and its implications for Plio-Pleistocene hunter-gatherers. *Journal of Anthropological Archaeology* 44:185-197.
41. Pop E*, et al.* (2016) Fires at Neumark-Nord 2, Germany: An analysis of fire proxies from a Last Interglacial Middle Palaeolithic basin site. *Journal of Field Archaeology*:1-15.
42. Mania D*, et al.* (2010) *Neumark-Nord. Ein interglaziales Ökosystem des mittelpaläolithischen Menschen* (Landesamt für Denkmalpflege und Archäologie Sachsen-Anhalt, Halle).
43. Roebroeks W*, et al.* (2021) Landscape modification by Last Interglacial Neanderthals. *Science Advances* 7(51):eabj5567.
44. Eichhorn G (1909) *Die paläolithischen Funde von Taubach in den Museen zu Jena und Weimar : Festschrift zum 350jährigen Jubiläum der Universität Jena* (Fischer, Jena).
45. Behm-Blancke G (1960) Altsteinzeitliche Rastplatxe im Travertingebiet von Taubach, Weimar, Ehringsdorf. *ALT-THURINGEN* 4:1-246.
46. Speth J (2017) Putrid Meat and Fish in the Eurasian Middle and Upper Paleolithic: Are We Missing a Key Part of Neanderthal and Modern Human Diet? *PaleoAnthropology* 2017:A1-A41.
47. Churchill S (2014) *Thin on the Ground: Neandertal Biology, Archeology and Ecology* (John Wiley & Sons, Ames, Iowa).
48. Vallverdú J*, et al.* (2010) Sleeping Activity Area within the Site Structure of Archaic Human Groups: Evidence from Abric Romaní Level N Combustion Activity Areas. *Current Anthropology* 51(1):137-145.
49. Hayden B (2012) Neandertal Social Structurel. *Oxford Journal of Archaeology* 31(1):1-26.
50. Duveau J, Berillon G, Verna C, Laisné G, & Cliquet D (2019) The composition of a Neandertal social group revealed by the hominin footprints at Le Rozel (Normandy, France). *Proceedings of the National Academy of Sciences* 116(39):19409-19414.
51. Mayoral E*, et al.* (2021) Tracking late Pleistocene Neandertals on the Iberian coast. *Scientific Reports* 11(1):4103.
52. Skov L*, et al.* (2022) Genetic insights into the social organization of Neanderthals. *Nature* 610(7932):519-525.
53. Lalueza-Fox C*, et al.* (2011) Genetic evidence for patrilocal mating behavior among Neandertal groups. *Proceedings of the National Academy of Sciences* 108(1):250-253.
54. Féblot-Augustins J (1999) Raw material transport patterns and settlement systems in the European Lower and Middle Palaeolithic: continuity, change and variability. *The Middle Palaeolithic Occupation of Europe*, eds Roebroeks W & Gamble CS (University of Leiden, Leiden), pp 193-214.
55. Roebroeks W, Kolen J, & Rensink E (1988) Planning depth, anticipation and the organization of Middle Palaeolithic technology: the 'archaic natives' meet Eve's descendants. *Helinium* 28:17-34.
56. Lee RB & DeVore I eds (1968) *Man the Hunter* (Aldine Publishing Company, Chicago).
57. Birdsell JB (1968) Some predictions for the pleistocene based on equilibrium systems among recent hunter-gatherers. *Man the Hunter*, eds Lee RB & DeVore I (Aldine, Chicago), pp 229-240.
58. Singh M & Glowacki L (2022) Human social organization during the Late Pleistocene: Beyond the nomadic-egalitarian model. *Evolution and Human Behavior* 43:418–431.
59. Bird DW, Bird RB, Codding BF, & Zeanah DW (2019) Variability in the organization and size of hunter-gatherer groups: Foragers do not live in small-scale societies. *Journal of Human Evolution* 131:96-108.
60. Migliano AB*, et al.* (2020) Hunter-gatherer multilevel sociality accelerates cumulative cultural evolution. *Science Advances* 6(9):eaax5913.

61. Kindler L*, et al.* (2020) The Last Interglacial (Eemian) lakeland pf Neumark-Nord (Saxony-Anhalt, Germany). Sequencing Neanderthal occupations, assessing subsistence opportunities and prey selection based on estimations of ungulate carrying capacities, biomass production and energy values. *Human behavioural adaptations to interglacial lakeshore environments.*, eds García-Moreno A, Hutson JM, Smith GM, Kindler L, Turner E, Villaluenga A, & Gaudzinski-Windheuser S (Propylaeum, Heidelberg), pp 67-104.
62. Sier MJ*, et al.* (2011) Direct terrestrial-marine correlation demonstrates surprisingly late onset of the last interglacial in central Europe. *Quaternary Research* 75(1):213-218.
63. Boyd R & Richerson PJ (2022) Large-scale cooperation in small-scale foraging societies. *Evolutionary Anthropology: Issues, News, and Reviews*:1-24.
64. Kahlke HD ed (1977) *Das Pleistozän von Taubach bei Weimar* (Akademie-Verlag, Berlin).
65. Guenther EW (1977) Die Backenzähne der Elefanten von Taubach bei Weimar. *Quartärpaläontologie* 5:389-408.
66. Laws RM (1966) Age criteria for the African elephant. *African Journal of Ecology* 4(1):1-37.
67. Jachmann H (1988) Estimating age in African elephants: a revision of Laws' molar evaluation technique. *African Journal of Ecology* 26(1):51-56.
68. Stansfield FJ (2015) A Novel Objective Method of Estimating the Age of Mandibles from African Elephants (*Loxodonta africana Africana*). *PLOS ONE* 10(5):e0124980.
69. Smuts MM & Bezuidenhout AJ (1993) Osteology of the thoracic limb of the African elephant (*Loxodonta africana*). *Onderstepoort J Vet Res* 60(1):1-14.
70. Bezuidenhout AJ & Seegers CD (1996) The osteology of the African elephant (*Loxodonta africana*): vertebral column, ribs and sternum. *The Onderstepoort journal of veterinary research* 63 2:131-147.
71. van der Merwe NJ, Bezuidenhout AJ, & Seegers CD (1995) The skull and mandible of the African elephant (*Loxodonta africana*). *Onderstepoort J Vet Res* 62(4):245-260.
72. Stanek GJ (2012) Die Gelenke der Hinterextremität des Afrikanischen Elefanten (*Loxodonta africana*). PhD-Thesis (Veterinärmedizinische Universität Wien, Vienna).
73. Lyman RL (1994) *Vertebrate Taphonomy* (Cambridge University Press, London).
74. Fernández-Jalvo Y & Andrews P (2016) *Atlas of Taphonomic Identifications. 1001+ Images of Fossil and Recent Mammal Bone Modification* (Springer, New York).
75. Haynes G & Klimowicz J (2015) Recent elephant-carcass utilization as a basis for interpreting mammoth exploitation. *Quaternary International* 359-360:19-37.
76. Marano F & Palombo MR (2013) Population structure in straight-tusked elephants: a case study from Neumark Nord 1 (late Middle Pleistocene?, Sachsen-Anhalt, Germany). *Bollettino Società Paleontologica Italiana* 52(3):207-218.
77. Discamps E & Costamagno S (2015) Improving mortality profile analysis in zooarchaeology: a revised zoning for ternary diagrams. *Journal of Archaeological Science* 58:62-76.
78. Haynes G (2017) Finding meaning in mammoth age profiles. *Quaternary International* 443:65-78.
79. Pop E, Bakels C, Kuijper W, Mücher H, & van Dijk M (2015) The Dynamics of Small Postglacial Lake Basins and the Nature of Their Archaeological Record: A Case Study of the Middle Palaeolithic Site Neumark-Nord 2, Germany. *Geoarchaeology* 30(5):393-413.
